# Exposure to and Burden of Major Non-Communicable Disease Risk Factors in Brazil and its States, 1990-2019: The Global Burden of Disease Study

**DOI:** 10.1590/0037-8682-0275-2021

**Published:** 2022-01-28

**Authors:** Caroline Stein, Maria Inês Schmidt, Ewerton Cousin, Deborah Carvalho Malta, Mohsen Naghavi, Patrícia Pereira Vasconcelos de Oliveira, Antonio Luiz Pinho Ribeiro, Bruce B. Duncan

**Affiliations:** 1 Universidade Federal do Rio Grande do Sul, Programa de Pós-Graduação em Epidemiologia, Porto Alegre, RS, Brasil.; 2 Universidade Federal do Rio Grande do Sul, Faculdade de Medicina, Departamento de Medicina Social, Porto Alegre, RS, Brasil.; 3 University of Washington, Institute for Health Metrics and Evaluation, Seattle, WA, United States .; 4 Universidade Federal de Minas Gerais, Escola de Enfermagem, Belo Horizonte, MG, Brasil.; 5 Ministério da Saúde, Departamento de Análise em Saúde e Vigilância de Doenças Não Transmissíveis, Brasília, DF, Brasil.; 6 Universidade Federal de Minas Gerais, Faculdade de Medicina, Departamento de Clínica Médica, Belo Horizonte, MG, Brasil.

**Keywords:** Risk factors, Noncommunicable diseases, Global Burden of Disease, Brazil

## Abstract

**INTRODUCTION:**

Non-Communicable Diseases (NCDs) have become the main cause of disease burden in Brazil. Our objective was to describe trends (1990 to 2019) in prevalence and attributable burden of five modifiable risk factors and related metabolic risk factors in Brazil and its states.

**METHODS:**

In Global Burden of Disease 2019 analyses, we described trends in prevalence of modifiable risk factors and their metabolic mediators as percentage change in Summary Exposure Value (SEV). We estimated deaths and disability-adjusted life years (DALYs) attributable to the risk factors.

**RESULTS:**

Age-adjusted exposures to alcohol [41.0%, Uncertainty Interval (UI): 24.2 - 63.4], red meat (61.2%, UI: 42.4-92.3), low physical activity (3.9%, UI: -5-17.5) and ambient particulate matter pollution (3.3%, UI: -48.9-128.0) have worsened. Those for smoking (-51.4%, UI: -54.7- - 47.8), diet low in fruits (-28.1%, UI: -39.1- -18.7) and vegetables (-19.6%, UI: -32.7 - -8.7), and household air pollution (-85.3%, UI: -92.9- -74.3) have improved. All mediating metabolic risk factors, except high blood pressure (0.7%, UI: -6.9-8.3), have worsened: BMI (110.2%, UI: 78.6-161.7), hyperglycemia (15.1%, UI: 9.3-21.2), kidney dysfunction (12.0%, UI: 8.4-17.2), and high LDL-c (11.8%, UI: 6.9-17.2).

**CONCLUSIONS:**

A variable pattern of progress and failure in controlling modifiable risk factors has been accompanied by major worsening in most metabolic risk factors. The mixed success in public health measures to control modifiable risk factors for NCDs, when gauged by the related trends in metabolic risk factors, alert to the need for stronger actions to control NCDs in the future.

## INTRODUCTION

The United Nations has established, as a Sustainable Development Goal (SDG): To ensure healthy lives and to promote well-being for all at all ages To this end, countries agreed to “By 2030, to reduce by one-third premature mortality from non-communicable diseases (NCDs) through prevention”^1^. The World Health Organization (WHO) established five modifiable risk factors - tobacco use, harmful use of alcohol, unhealthy diet, physical inactivity, and air pollution - as the primary focus to decrease the burden of NCD^2^ ^-^ ^6^. 

The Brazilian Ministry of Health launched in 2011 its Strategic Action Plan to Tackle NCDs 2011-2022 to confront the challenge of the NCDs^7^. The plan, following WHO guidance of that time, focuses on decreasing the prevalence of the risk factors tobacco use, harmful use of alcohol, unhealthy diet, and physical inactivity^8^. A new plan for the years 2022-2030 is expected^9^. Knowledge of current levels of the main NCD risk factors and their trends for over recent decades is key to gauging the success of these efforts and to planning future action. This is especially true as Brazil is not currently on track to achieve the Sustainable Development Goal of decreasing premature NCD mortality^10^. Thus, our objective is to describe levels and trends of exposure and burden of the five main modifiable NCD risk factors and of the metabolic risk factors that mediate their burden in Brazil and its states from 1990 to 2019.

## METHODS

We used data from the Global Burden of Disease (GBD) Study 2019, applying standard GBD methods^11^. The principal metrics employed are the Summary Exposure Value (SEV), and rates of deaths and disability-adjusted life-years (DALYs). We employed the SEV, the Global Burden of Disease´s more sophisticated alternative to prevalence, to estimate exposure to risk factors. As explained in greater detail elsewhere^12^ ^,^ ^13^, the SEV measures population exposure to risk factors by estimating the prevalence of different levels of the factor, each weighted by the size of that level´s contribution to disease burden. For example, in two populations with equal prevalence of current smokers, the SEV of smoking would be higher in the one in which smokers on average smoked more cigarettes. A decline in SEV over time indicates reduced exposure; an increase in SEV, increased exposure. DALYs are the sum of years lost due to premature death and years lived with disability; this metric is also defined as years of healthy life lost. The risk factors in question are grouped within three broad categories by the GBD:

Behavioral: smoking, alcohol use, low physical activity, and dietary risks (the main ones being consumption high in sodium or red meat, and low in whole grains, fruits, and vegetables).

Environmental: ambient particulate matter and household air pollution.

Metabolic: high systolic blood pressure, high fasting plasma glucose, high body-mass index (BMI), high low-density lipoprotein-cholesterol (LDL-c) and kidney dysfunction. 

The GBD definitions for these risk factors are presented in  Supplementary Material Table 1.

The GBD utilizes the Socio-demographic Index (SDI)^14^, to characterize level of development. 

Statistical analyses are primarily descriptive. To facilitate comparison of secular trends in the exposure (SEV) to differing risk factors, we express the percentage change of each from its 1990 baseline level. To describe exposure over the life course to risk factors, we standardized SEV values to age 50, dividing the original value at each age by that at age 50 and multiplying by 100. For example, if the original SEV at age 50 were 20, and that at age 70 were 40, the standardized SEV we present for age 70 would be 200. Values were calculated for each 5-year age strata.

## RESULTS

**FIGURE 1:**
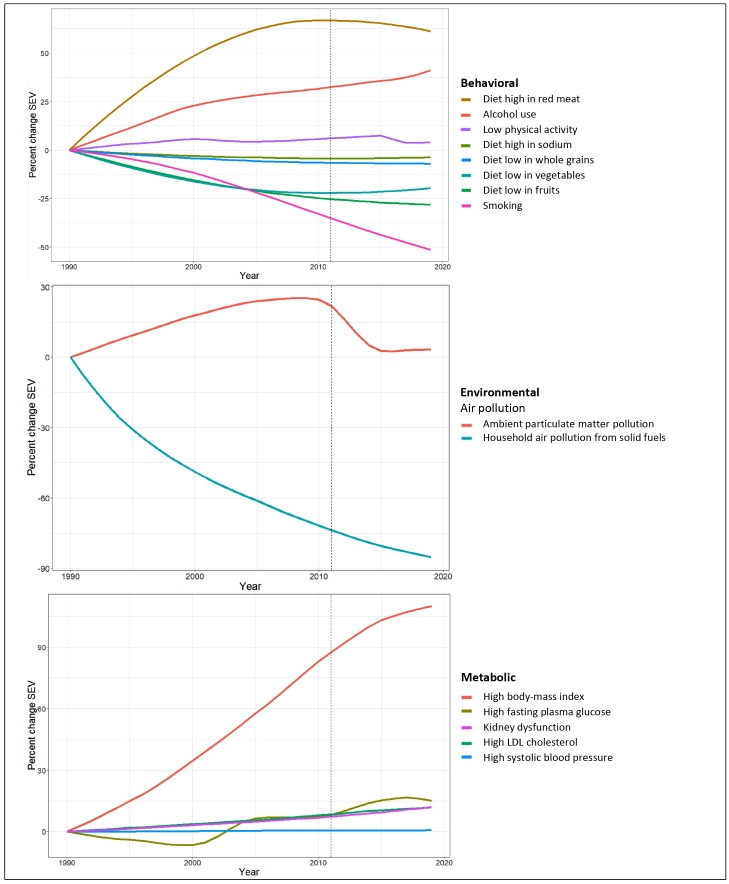
Trends in exposure to risk factors, as measured by their age-standardized summary exposure values (SEVs), both sexes, from 1990 to 2019, Brazil. **Top:** Behavioral, **Middle:** Environmental, **Bottom:** Metabolic.

Figure 1 shows a variable pattern of change in NCD risk factors in Brazil from 1990 to 2019. Among the WHO-priority modifiable behavioral risk factors (top panel), large decreases occurred for smoking [-51.4%, Uncertainty Interval (UI): -54.7 - -47.8], and two components of dietary risk, diet low in fruits (-28.1%, UI: -39.1 - -18.7) and in vegetables (-19.6%, UI: -32.7 - -8.7), and large increases occurred for alcohol consumption (41.0%, UI: 24.2 - 63.4) and the dietary risk component, a diet high in red meat (61.2%, UI: 42.4 - 92.3). Minor changes occurred for the remaining WHO-priority risk factors, including low physical activity (3.9%, UI: -5.0 - 17.5). As seen from the middle panel, air pollution also varied, household air pollution from solid fuels decreasing markedly (-85.3%, UI: -92.9 - -74.3), and ambient particulate matter pollution increasing (3.3%, UI: -48.9 - 128.0). The pattern of change observed for metabolic risk factors (lower panel) was almost uniformly unfavorable, led by that of population exposure of high BMI, with an increase of (110.2%, UI: 78.6 - 161.7), followed by high fasting plasma glucose (15.1%, UI: 9.3 - 21.2), kidney dysfunction (12.0%, UI: 8.4 - 17.2), high LDC-c (11.8%, UI: 6.9 - 17.2), and high systolic blood pressure (0.7%, UI: -6.9 - 8.3). 

The trends in many risk factors have been generally consistent in both direction and velocity of change over the whole period. After the 2011 launch of the Brazilian plan to confront NCDs (indicated by a vertical line in the figures), some improvements were seen. Excess consumption of red meat decreased as did low physical activity. Improvement in vegetable consumption stabilized and, more recently, reversed. Ambient particulate matter pollution improved notably. In parallel, all metabolic risk factors have continued to worsen. Over more recent years, slightly lower increases for high BMI and high fasting plasma glucose can be seen. 

Table 1 compares trends of several of these risk factors since 2010 with trends in metrics used to evaluate success by the Ministry of Health, in the Vigitel Phone Survey^9^. Changes regarding smoking and alcohol consumption were similar. A lesser worsening high BMI was found in the GBD estimation, in which a higher 2010 baseline than that of Vigitel was present. While the Vigitel measure of leisure time physical activity, the indicator adopted by Brazil´s 2011 Plan, showed marked improvement, the GBD SEV of capturing overall physical activity in all dimensions, was basically unchanged. Vigitel measures of fruit and vegetable consumption improved considerably more than GBD SEVs. 

**TABLE 1:** Changes in the prevalence of selected risk factor as estimated from Vigitel compared to change in similar exposures from 2010-2019 as estimated by the age-standardized GBD summary exposure values (SEVs).

	Ministry of Health			GBD 2019		
Risk factor	Prevalence	Prevalence	Change in	SEV 2010	SEV 2019	Change in SEV
	2010	2019	Prevalence	(Age-std)	(Age-std)	(%)
	(%)	(%)	(%)			
Smoking	14.1	9.8	-30.5	12.79 (11.09 - 14.73)	9.26 (8.03 - 10.76)	-28 (-30 - -25)
Alcohol use	18.1	18.8	3.9	8.66 (6.34 - 11.38)	9.27 (6.44 - 12.51)	7 (-4 - 18)
High BMI	15.1	20.3	34.4	27.19 (21.96 - 34.87)	31.22 (25.51 - 39.47)	15 (10 - 21)
Active in leisure time	30.5	39.0	27.9	-	-	-
Low physical activity	-	-	-	12.71 (8.01 - 19.35)	12.49 (7.75 - 19.2)	-2 (-6 - 2)
Diet adequate in fruits and vegetables	19.5	22.9	17.4	-	-	-
Diet low in fruits	-	-	-	34.02 (24.02 - 45.45)	32.48 (22.82 - 43.80)	-5 (-13 - 5)
Diet low in vegetables	-	-	-	61.87 (41.51 - 80.98)	63.84 (43.79 - 81.60)	3 (0 - 8)
Diet high in sodium	-	-	-	29.06 (7.26 - 52.83)	29.23 (7.51 - 52.70)	1 (-15 - 18)

**FIGURE 2:**
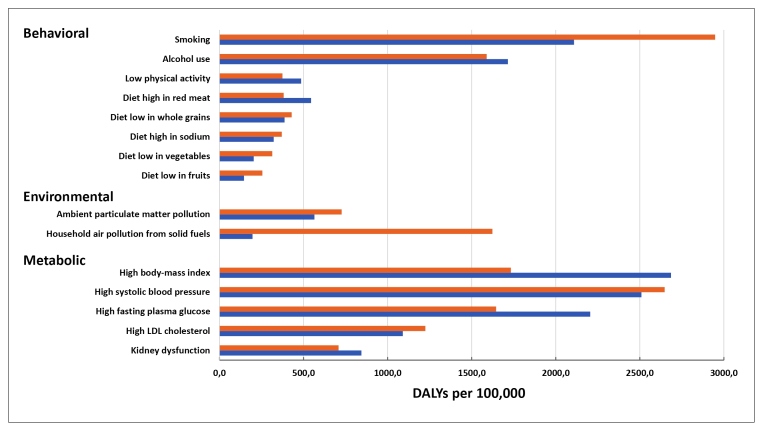
All-cause DALYs per 100,000 attributable to risks factors, 1990-2019, all-ages, both sexes, Brazil. **Orange bars:** 1990, **blue bars:** 2019.

Figure 2 presents levels of disease burden in 1990 and 2019, expressed in crude, all-cause DALYs, due to these risk factors. In 2019, among the modifiable behavioral risk factors, the largest burden was attributable to smoking (2,108 DALYs/100,000 population; UI: 1,967 - 2,527) following by that of alcohol use (1,715 DALYs/100,000; UI: 1,481 - 1,951). While the burden due to smoking decreased from 1990, that of alcohol and low physical activity increased. The burden from the dietary risk factors, except for that for red meat, decreased. DALYs due to ambient and especially household air pollution from solid fuels both decreased over the period. The burden due to metabolic risk factors increased for high BMI, high glucose, and kidney disfunction, but decreased for high systolic blood pressure and high LDL-c. High BMI showed the largest increase, growing 54.9%, to 2,685 DALYs/100,000 (UI: 1,937 - 3,480).

**FIGURE 3:**
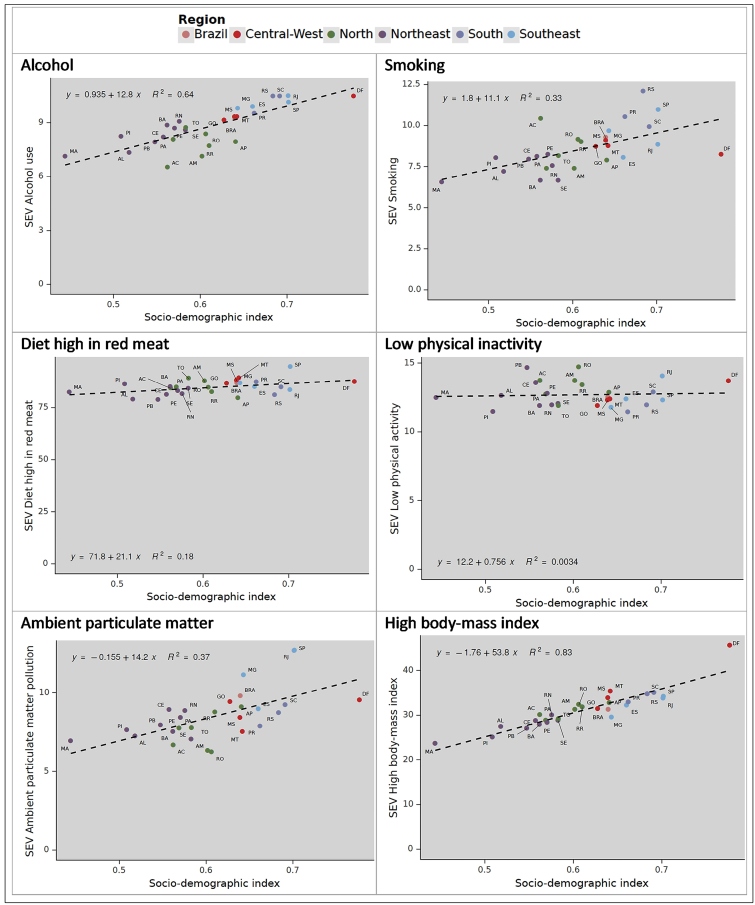
Panel of relationship between summary exposure values (SEVs) and Socio-demographic Index (SDI) 2019, both sexes, age-standardized, Brazil and its states.

Figure 3 presents the distribution of exposure (SEV) to the main modifiable risk factors and one metabolic risk factor - high body mass index - in 2019 for Brazilian states characterized by their SDI. Exposure to most, but not all of these risk factors, and most notably for high BMI, increased with increasing SDI.  Supplementary Material Figure 1 shows similar plots, now demonstrating the change in risk factor exposure (SEV) from 1990-2019 for Brazilian states, again displayed by their level of development. Most risk factors showed greater increases in exposure in low SDI states.

**FIGURE 4:**
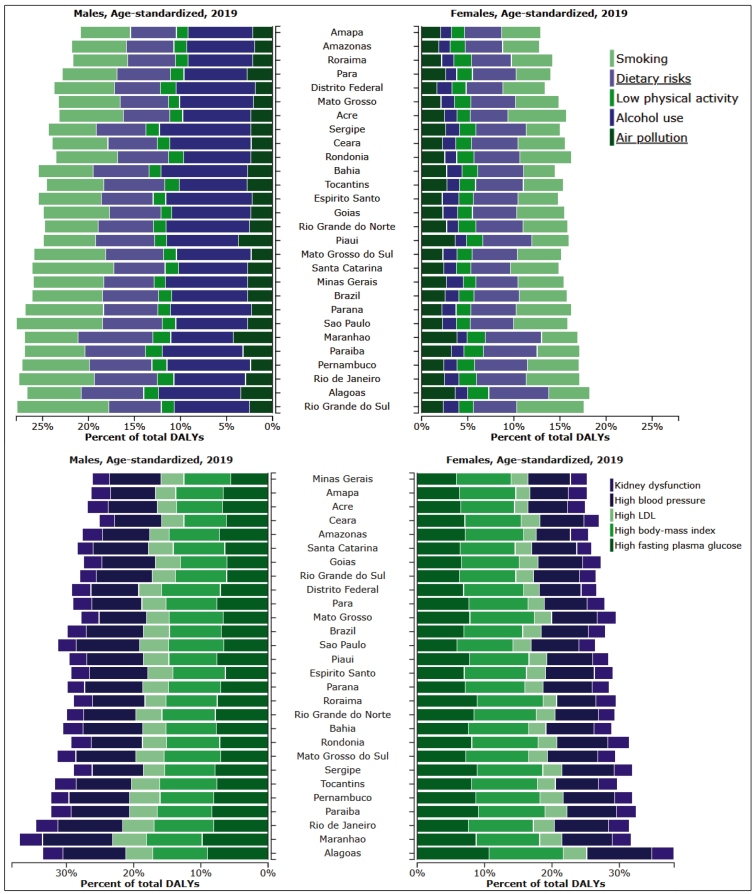
All-cause DALYs (%) attributable to the five main modifiable risk factors (top panel) and their metabolic mediators (bottom panel), 2019, females (right bars) and males (left bars), age-standardized, Brazil and its states. See the specific values of percent of total DALYs at: http://ihmeuw.org/5fa9 and http://ihmeuw.org/5faa.

Figure 4 (top panel) presents the percent of 2019 age-standardized all-cause DALYs attributable to the five main risk factors separately for men and women for Brazil and its states. When considering Brazil as a whole, alcohol use and smoking were the behavioral risk factors most responsible for all-cause DALYs among males and smoking and dietary risks among females. The rankings were generally similar across states, the smallest burden tended to be for states from the North and Northeast, with Rio Grande do Sul and Alagoas being the states with greatest burden. Considering the metabolic risk factors (Figure 4, bottom panel), the main factors causing burden were high systolic blood pressure, high glucose and high BMI for both males and females. States with a higher burden were frequently from the Northeast. Alagoas showed the greatest burden, and Amapá and Minas Gerais the least.

Supplementary Material Figures 2, 3, 4, 5 shows changes in the extent of exposure to these risk factors over the adult life course, as estimated by standardized SEVs. In these graphs, the SEV at age 50 for each risk factor is set to 100 and then used as a reference for the standardized SEV values at other ages. Ages in which the SEV, so presented, is above 100 represent periods over the lifespan of relatively greater risk exposure; those with values below 100, lesser exposure. Alcohol use is greatest in young adults, low physical activity in older adults, and smoking exposure rises throughout adulthood to its greatest level in the 60 to 70 age range ( Supplementary Material Figure 2). Exposure to dietary risks tends to be greater in young adulthood, and an increase in the exposure to a low vegetable and high sodium consumption is seen after age 50 ( Supplementary Material Figure 3). Exposure to ambient air pollution is relatively low throughout childhood and adolescence, and then becomes uniformly higher in adulthood ( Supplementary Material Figure 4). With respect to metabolic risk factors ( Supplementary Material Figure 5), major exposure to high BMI begins earlier than the rest, rises to age 50 and then tapers. The other metabolic risks rise throughout the adult age span, most notably so for high fasting plasma glucose and for kidney dysfunction. High systolic blood pressure shows a similar, but slightly less sharp upward inclination over age. Low LDL-c presents a gradual increase to age 65 with posterior stabilization. 

Rankings of risk factors as the cause of crude, all-cause DALY rates and death rates ( Supplementary Material Figure 6) shows a relative ascension from 1990 to 2019 of all NCD risk factors, except for air pollution and tobacco when burden is expressed both in terms of deaths and DALYs. 

In terms of DALYs, in 1990 child and maternal malnutrition was the principal risk factor, but by 2019 it had fallen to 7th place. Nine of the 10 most important risk factors were the five WHO-priority modifiable factors here analyzed, or their related metabolic risk factors. Low physical activity occupied the 12th position. High BMI and high systolic blood pressure were the two main causes of burden. 

In terms of deaths, the rankings and their changes were similar, though the burden of low physical activity rose over time while that of dietary risks declined slightly.

## DISCUSSION

The changes in exposure to and burden from the five principal modifiable NCD risk factors over the last three decades in Brazil have been mixed. They have been accompanied by considerable worsening in exposure to and resultant burden from several metabolic mediators of NCDs. Given greater progress in controlling communicable diseases/maternal and child conditions, almost all of the top-ranked causes of overall disease burden in 2019 are these NCD risk factors and their metabolic mediators. The burden attributable to them is generally uniform across Brazilian states.

Few summaries exist of trends in NCD risk factors over the past decade. The GBD, with its systematic approach to gathering such data, offers an important resource to track trends in these factors over time in countries around the world. For Brazil, one important role of the GBD, given its different calculation of population denominators, its use of a greater number of data sources, and its extensive methodologic adjustments to deal with the imperfections of vital statistics, is to validate the more straightforward Ministry of Health estimates, and to provide information where standard Ministry surveillance is lacking. 

Evaluation of risk factors for which the 2011 Plan presented goals using GBD data presents a picture somewhat less favorable than that when they are evaluated through Ministry surveys. Though Vigitel^9^ suggests only a 3.9% increase in harmful alcohol consumption, the GBD estimates that total alcohol consumption in drinkers has increased 7% (UI: -4 - 18). While Vigitel shows a 27.9% increase in leisure time physical activity, the GBD, evaluating overall physical activity, shows only a -2% (UI: -6 - 2) change in low physical activity (Table 1). Evaluating a somewhat broader list of dietary risks, the GBD suggests that fruits and vegetables consumption has not increased as much as suggested by Vigitel estimates, and that red meat consumption, which it considers the main dietary risk, after peaking in 2010, has decreased 3.0% (UI: -7 - -1) since then. Smoking estimates are similar, and in the absence of Ministry tracking, the GBD suggests that indoor air pollution has decreased 48% (UI: -66 - -26) while ambient outdoor particulate matter pollution has decreased 17% (UI: -34 - 1), and that high sodium consumption has remained stable over the period. In this regard, the 2014-15 Laboratory sub-study of the 2013 National Health Survey showed a mean salt consumption of close to 10g/day (vs. the Plan´s goal for that year of ~8g/day), with 96.4% of the Brazilian population consuming >5 g per day, that is, above the goal established in Brazil´s Plan for 2022^15^.

The direct relationship found between risk factor exposure and developmental level (SDI) may be a factor making future risk factor control more difficult as all states will likely move to a higher SDI over time. That changes from 1990 in exposure to several risk factors were greatest in states with lower SDIs suggests that social disparities in prevalence of these risk factors will also likely worsen over time.

Trends in deaths and DALYs caused by these risk factors generally parallel those of risk factor exposure. Large decreases over time in burden were seen for smoking and indoor air pollution, major worsening in burden for high BMI and hyperglycemia. The more favorable trends in risk factor burden than for risk factor exposure can be explained by what the GBD, for lack of a better term, calls “risk-deleted” changes. These are changes in other factors, the most prominent among them improvement in access to and quality of health care. As health care, especially for cardiovascular disease, has expanded and improved, its burden is now less for any given risk factor level than in the past. Improvement in other, as yet undescribed or poorly described, risk factors may also be at work.

In the GBD analyses, considering the five WHO-priority modifiable risk factors, smoking presents one of the most favorable trends, a result of strong, population-based, fiscal and legislative approaches requiring little individual agency. Control of alcohol stands out among the risk factors with unfavorable trends. Considering the metabolic factors, control of increasing adiposity has been the principal failure of recent decades. This is particularly important as increasing adiposity worsens the profile of all the remaining metabolic factors, especially hyperglycemia, for which exposure and burden have also seen large increases over the period. It is worth noting, with respect to these metabolic factors, that clinical actions also impact on their prevalence. This is especially true for hypertension and high LDL-c, for which inexpensive, highly effective drugs with few side effects are currently available. 

Though much remains to be learned about the underlying causes of changes in the occurrence of these diseases^16^, estimates show that decreasing risk factors, especially smoking and alcohol, could play a major role in achieving the SDG goals^17^. Thus, these trends in terms of future NCD burden are worrisome. As important lags exist between risk factor exposure and disease onset, current unfavorable trends in exposure to alcohol, overall physical activity, ambient air pollution, and the metabolic risk factors portend great difficulty in reaching the announced SDG goal of decreasing premature NCD mortality by one-third by 2030^1^. Additionally, the COVID-19 pandemic has disrupted society and healthcare worldwide, potentially reversing decades of improvement in health. Ninety percent of countries report disruptions to essential health services, many of which deal with control of NCD risk factors such as hypertension^18^. More importantly, social distancing has been reported to increase sedentary activities, decrease physical activity, and increase consumption of ultraprocessed foods, alcohol and tobacco among Brazilian adults^19^. All of these trends, likely to be accompanied by weight gain, will have negative effects on the prevalence of cardiometabolic risk factors. 

Historically, successful disease control has generally been achieved through public health measures based on the identification and elimination of their risk factors. Yet, despite widespread recognition of the causal relationships underlying the NCDs, efforts for their control through decreasing their risk factors have received minimal resources viz-a-viz those allocated to disease treatment, and, as presented here, has achieved limited success. 

Population-based strategies requiring little individual agency, when implemented, have been shown to be the most effective options to NCD control^20^. For example, the successful decrease in smoking in Brazil has resulted from concerted government interventions since the 1980s^21^ based on taxation and on legislation prohibiting smoking in public places and its advertising. The 2011 tobacco-free environment law, regulated in 2014, stands out as a breakthrough in the country's regulatory policy^22^. 

Reduction in household air pollution from solid fuels, to the extent that it is due to improved living conditions resulting from public policies such as the *Bolsa Família* cash transfer program^23^, could be considered another success of the, population-based, low individual agency approach.

Barriers to greater implementation of this type of strategy are mainly political, not unlike those of stimulating greater mask use and social distancing to prevent COVID-19. Society must come to recognize the disease burden caused by unregulated or minimally regulated exposure to risk factors and must believe that government interventions are justified. However, the setting of NCD risks is more complicated than that of SARS-CoV-2 control, as interventions are frequently opposed by powerful, corporate interests and lobbying.

Until society assumes the posture that greater interventions by government (e.g. advertising restriction, labelling and taxes for unhealthy foods^24^, incentives to increase the availability of healthy foods, investments facilitating physical activity, greater restrictions on smoking, greater control of alcohol advertising and sales) are justified, and the workings of government can be made to respond to this posture, it is hard to imagine that trends in many of these risk factors will improve appreciably. 

Revision of public policies has recently been undertaken in the development of the Strategic Action Plan to Tackle NCDs in Brazil^7^. Hopefully the Plan, with its multiple planned actions to control risk factors and diseases both at the population and clinical levels, and which includes the addition of two new nutritional goals related to ultra-processed foods, mention of some of the WHO “best buys”^25^ ^,^ ^26^, incorporation of air pollution as a major risk factor meriting action, and more aggressive goals for childhood and adolescent obesity, physical activity and fruit and vegetable consumption, will lead to changes in the metabolic risk factors and help maintain the continued age-standardized decline in premature NCD mortality.

We additionally have found that trends in the WHO-priority modifiable risk factors (e.g. improvement in many dietary risks and a relative stability in overall physical activity) correlate poorly with trends in metabolic risk factors they presumedly cause (e.g. markedly higher exposure to high BMI and hyperglycemia). This raises the issue as to whether risk factor targets, as currently defined, are sufficient to gauge the resultant effectiveness of actions whose ultimate objective is to decrease the metabolic risks. Greater understanding of the genesis of these metabolic risks in Brazilian society is necessary, and additional targets for interventions should be sought. In this regard, the addition of nutritional targets related to ultra-processed foods, greater consumption of which has been shown to be associated with weight gain and obesity^27^ ^,^ ^28^ in the proposed Brazilian plan is laudatory. 

It is important to recognize limitations in our study, principally related to the availability and quality of the primary data included in the GBD estimates^11^. However, the sophistication of GBD analytic approaches taken to overcome these limitations, such as the detailed approach of redistribution of ill-defined causes of death, coupled with constant review and updating of inputted data by the GBD Brazilian Network, make GBD analyses the best currently available. An additional limitation is the inability, to date, to evaluate within-population disparities in the social determinants of health when using the GBD framework of analyses.

In conclusion, exposure to and burden from smoking, indoor air pollution and many of the principal dietary factors for NCDs have decreased in Brazilian society over the past three decades. Low overall physical activity has increased slightly. In contrast and more importantly, exposure to alcohol consumption, red meat, ambient air pollution and all of the relevant metabolic risk factors, especially high BMI and hyperglycemia, have worsened over the last thirty years, with the metabolic risks continuing to do so over the past decade. Brazil is not achieving its Sustainable Development Goal to reduce premature NCD mortality by one-third by 2030. Persistent and more aggressive public interventions, particularly those achieved through population-based actions requiring little individual agency, are needed to improve trends in major NCD risk factors. To implement these interventions, strengthened social and political support will be necessary.
