# Supplementary material for: Exposure to and Burden of Major Non-Communicable Disease Risk Factors in Brazil and its States, 1990-2019: The Global Burden of Disease Study
Source: Rev Soc Bras Med Trop. 2022 Jan 28;55(Suppl 1):e0275-2021. doi: 10.1590/0037-8682-0275-2021 (PMC9022946; doi:10.1590/0037-8682-0275-2021)
Supplement: Supplementary file 1 [file 1678-9849-rsbmt-55-e0275-2021-supp1.pdf]

Supplementary Table 1. GBD risk factors definitions.

**Risk factor (Age range)**	**GBD definition**
**Behavioral**	
Diet high in red meat (Age 25+)	Any intake (in grams per day) of red meat including beef, pork, lamb, and goat but excluding poultry, fish, eggs, and all processed meats.
Diet high in sodium (Age 25+)	Average 24-hour urinary sodium excretion (in grams per day) greater than 1-5 grams.
Diet low in vegetables (Age 25+)	Average daily consumption (in grams per day) of less than 280-320 grams of vegetables, including fresh, frozen, cooked, canned, or dried vegetables and excluding legumes and salted or pickled vegetables, juices, nuts and seeds, and starchy vegetables such as potatoes or corn.
Diet low in fruits (Age 25+)	Average daily consumption (in grams per day) of less than 310-340 grams of fruit including fresh, frozen, cooked, canned, or dried fruit, excluding fruit juices and salted or pickled fruits.
Diet low in whole grains (Age 25+)	Average daily consumption (in grams per day) of less than 140-160 grams of whole grains (bran, germ, and endosperm in their natural proportion) from breakfast cereals, bread, rice, pasta, biscuits, muffins, tortillas, pancakes, and other sources.

Alcohol use (Age 5+)	Grams per day of pure alcohol consumed among current drinkers.
Smoking (Age 30+)	The prevalence of current smoking and the prevalence of former smoking using data from cross-sectional nationally representative household surveys. Current smokers are individuals who currently use any smoked tobacco product on a daily or occasional basis. Former smokers as individuals who quit using all smoked tobacco products for at least six months, where possible, or according to the definition used by the survey.
Low physical activity (Age 25+)	Physical activity performed by adults older than 25 years of age, for duration of at least ten minutes at a time, across all domains of life. The frequency, duration and intensity of activity is used to calculate total metabolic equivalent-minutes per week.
**Environmental**	
Ambient particulate matter (Early neonatal +)	The population-weighted annual average mass concentration of particles with an aerodynamic diameter less than 2.5 micrometers ($PM_{2.5}$) in a cubic meter of air. This measurement is reported in $\mu g/m^3$.
Household air pollution (Early neonatal +)	From solid fuels (HAP) is estimated from both the proportion of individuals using solid cooking fuels and the level of PM2.5 air pollution exposure for these individuals. Solid fuels in our analysis include coal, wood, charcoal, dung, and agricultural residues.
**Metabolic**	
High SBP	Brachial systolic blood pressure in TMREL= 110-115 mmHg.

(Age 25+)	
High FPG (Age 25+)	Measured as the mean FPG in a population, where FPG is a continuous exposure in units of mmol/L. Since FPG is along a continuum, high FPG is defined as any level above the TMREL, which is 4.8-5.4 mmol/L.
High BMI (Age 1 +)	For adults (ages 20+) is defined as BMI greater than 20 to 25 kg/m^2. High BMI for children (ages 1–19) is defined as being overweight or obese based on International Obesity Task Force standards.
High LDL cholesterol (Age 25+)	Blood concentration of low-density lipoprotein (LDL) cholesterol, above TMREL of 27-50 mg/dl.
Kidney dysfunction (Age 25+)	Estimated glomerular filtration rate (eGFR) less than 60 ml/min/1.73m^2 or albumin to creatinine ratio (ACR) greater than or equal to 30 mg/g.

TMREL: theoretical minimum risk level
