# Supplementary material for: Exposure to and Burden of Major Non-Communicable Disease Risk Factors in Brazil and its States, 1990-2019: The Global Burden of Disease Study
Source: Rev Soc Bras Med Trop. 2022 Jan 28;55(Suppl 1):e0275-2021. doi: 10.1590/0037-8682-0275-2021 (PMC9022946; doi:10.1590/0037-8682-0275-2021)
Supplement: Supplementary file 2 [file 1678-9849-rsbmt-55-e0275-2021-supp2.pdf]

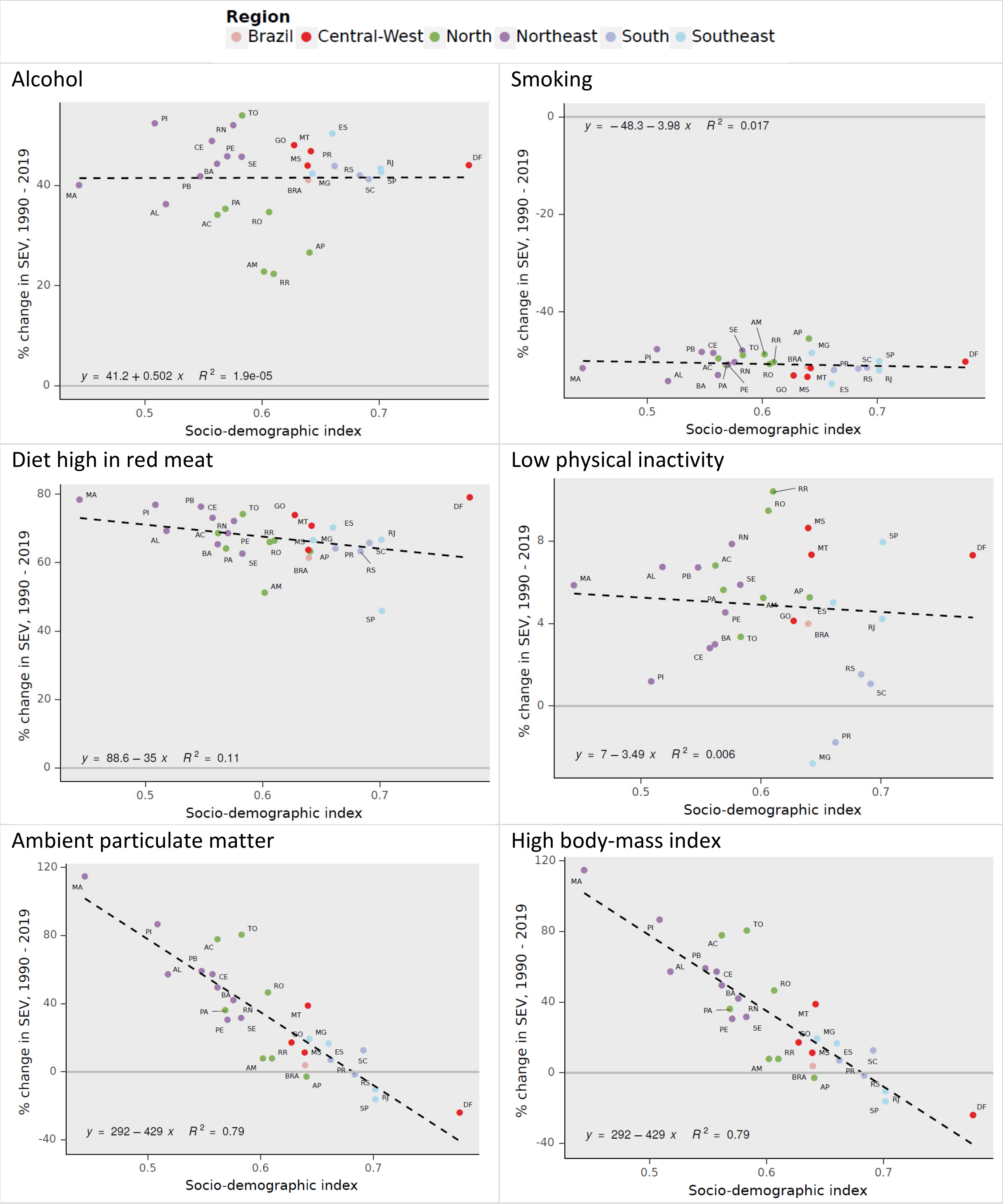

Supplementary Figure 1. Panel of relationship between the percent change of summary exposure values (SEV) from 1990 to 2019 and the Socio-demographic Index (SDI) 2019, both sexes, age-standardized, Brazil and its states.
