# Supplementary material for: Exposure to and Burden of Major Non-Communicable Disease Risk Factors in Brazil and its States, 1990-2019: The Global Burden of Disease Study
Source: Rev Soc Bras Med Trop. 2022 Jan 28;55(Suppl 1):e0275-2021. doi: 10.1590/0037-8682-0275-2021 (PMC9022946; doi:10.1590/0037-8682-0275-2021)
Supplement: Supplementary file 6 [file 1678-9849-rsbmt-55-e0275-2021-supp6.pdf]

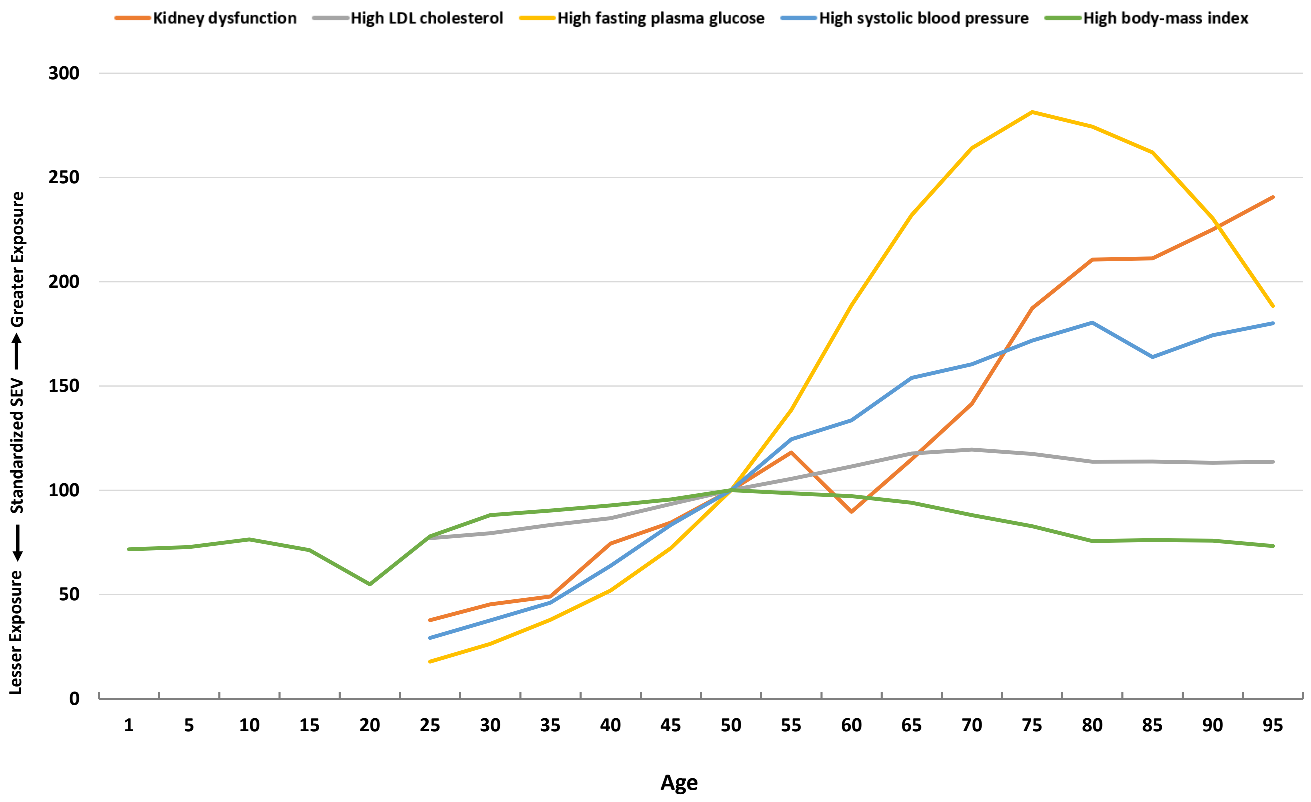

Supplementary Figure 5. Summary exposure values (SEVs) for metabolic risk factors showing the ages during which they are most present. The SEV at age 50 for each risk factor is set to 100 and then used as a reference for the calculations at other ages.
