# Supplementary material for: Exposure to and Burden of Major Non-Communicable Disease Risk Factors in Brazil and its States, 1990-2019: The Global Burden of Disease Study
Source: Rev Soc Bras Med Trop. 2022 Jan 28;55(Suppl 1):e0275-2021. doi: 10.1590/0037-8682-0275-2021 (PMC9022946; doi:10.1590/0037-8682-0275-2021)
Supplement: Supplementary file 7 [file 1678-9849-rsbmt-55-e0275-2021-supp7.pdf]

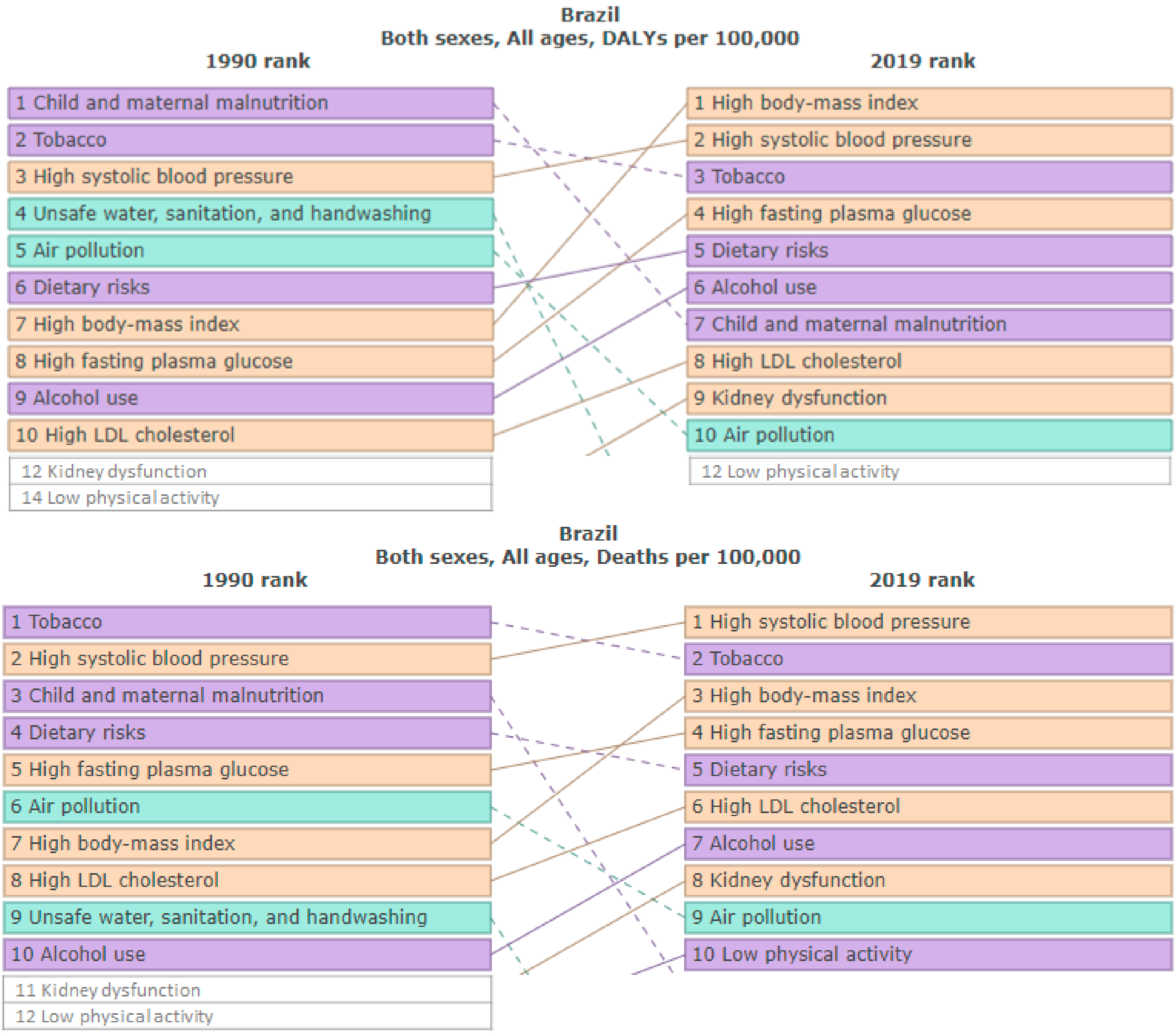

Supplementary Figure 6. Ranking of top 10 risk factors in terms of the crude, all-cause DALYs and deaths attributable to them, Brazil 1990-2019. Purple=behavioral, Orange=metabolic, Blue=environmental. See the specific values of percent change and rates at:
http://ihmeuw.org/5ds3 (DALYs) and http://ihmeuw.org/5fa8 (Deaths).
